# Developing and Validating Measures of Structural Ableism to Improve Health Outcomes for the Disability Community: Protocol for a Mixed Methods Study

**DOI:** 10.2196/86976

**Published:** 2026-03-13

**Authors:** Rupa S Valdez, Bonnielin K Swenor, Franz Castro, Caroline Cerilli, Bailey A Middleton, Kara S Fitzgibbon, Ashley Shew, Varshini Varadaraj, Hari Srinivasan, Diana Cejas

**Affiliations:** 1 Department of Systems and Information Engineering, Public Health Sciences University of Virginia Charlottesville, VA United States; 2 Disability Health Research Center Johns Hopkins University Baltimore, MD United States; 3 School of Nursing Columbia University Irving Medical Center New York, NY United States; 4 Center for Survey Research University of Virginia Charlottesville, VA United States; 5 College of Liberal Arts and Human Sciences Virginia Tech Blacksburg, VA United States; 6 Brain Institute Vanderbilt University Nashville, TN United States; 7 Department of Neurology University of North Carolina at Chapel Hill Chapel Hill, NC United States

**Keywords:** structural ableism, ableism, disability, health equity, health disparities, disability health, minority health

## Abstract

**Background:**

Structural ableism, defined as the processes, policies, and institutions that privilege able-bodied people over disabled people, is a root cause of health inequalities faced by the disability community. Unlike other forms of structural oppression, there are currently no adequate measurements for structural ableism and its impacts. Therefore, a necessary first step to addressing health inequities that impact the disability community is to create validated measures of structural ableism.

**Objective:**

This paper outlines the methods of an ongoing project that aims to develop and validate measures of structural ableism. The resulting measures will facilitate the identification of relationships between structural ableism and health outcomes at both an individual and community level.

**Methods:**

This project will take place across 3 phases. In Phase I, we will characterize the multiple factors that comprise the construct of structural ableism. We will begin by analyzing texts that discuss historical events, ideologies, and the lived experiences of disabled people to inform our understanding of contemporary dimensions of structural ableism. Simultaneously, key informant interviews with advocates and activists from the disability rights and disability justice movements will be conducted to further characterize the dimensions of structural ableism. In Phase II, the findings of Phase I will be used to inform the creation of an individual-level measure of structural ableism. Phase III will result in community-level measures of structural ableism, which will be developed using community-engagement studios with members of the disability community and publicly available datasets. This phase will build on the findings of the first 2 phases. Our methods purposefully include disabled people across all phases of this work, with a focus on maximizing the diversity of disability perspectives by including people across disability types and intersecting identities (eg, race and ethnicity, gender identity, geographic location, and other identities and demographics). Most importantly, our approach is deeply community-informed, drawing on multiple community partnerships from local and national organizations, a diverse advisory committee of disabled activists, advocates, and scholars, as well as researchers with expertise in developing measures of structural oppression, such as structural racism.

**Results:**

This project was funded in August of 2024. As of October 2025, our team has read more than 50 texts as part of our historical and policy analysis of the factors that characterize structural ableism. We plan to complete our characterization of structural ableism in the spring of 2026, with individual-level measures of structural ableism being developed by the Winter of 2028 and community-level measures created by the Winter of 2029.

**Conclusions:**

The measures developed by this work will lay the foundation for identifying and evaluating novel interventions aimed at dismantling structural ableism, which should be cocreated with the disability community.

**International Registered Report Identifier (IRRID):**

DERR1-10.2196/86976

## Introduction

The disability community was designated as a health disparity population by the National Institute on Minority Health and Health Disparities in 2023 [1], a recognition that disabled people face health disparities such as higher rates of mortality [2] and cancer [3], more severe outcomes of infectious diseases such as COVID-19 [4], poorer mental health [5], 4 times higher rates of victimization [6], and greater poverty [7,8] compared to nondisabled people. These disparities compound for disabled people who are part of other health disparity populations [7-10].

While research has examined how ableism within health care systems and from health professionals can lead to health disparities [11], there is a lack of knowledge on the greater impact of structural ableism on health outcomes. Structural ableism, which can be defined as “a complex system of hierarchical and discriminatory processes, policies, and institutions that privilege and prefer able-bodied people, fail to represent or meaningfully include disabled persons’ voices, and are grounded in a network of ableist beliefs and practices that maintain and reproduce unequal outcomes for disabled people and benefit able-bodied people” [12], is a force embedded in our society that produces poor health outcomes for disabled people that often go unnoticed and understudied.

With a PubMed [13-25] search of “structural ableism” in April 2025 yielding only 13 papers, the conceptual clarity of structural ableism within the health sciences remains in its infancy, and measures remain inadequate. Achieving conceptual clarity is essential for identifying the factors that comprise structural ableism and is a foundational step for understanding and addressing the ways in which structural ableism shapes health outcomes for disabled people [26]. Validated measures are essential for analyzing and addressing the mechanisms and pathways linking structural ableism to health outcomes for disabled people [14,27].

An important aspect of this work is recognizing that there are certain disability types that historically have been excluded from conversations about disability. Although, for instance, the definition above is among the most current, we acknowledge that some of the terminology (such as “able-bodied”) used to refer to people without disabilities may imply an overemphasis on physical disabilities. With this project, we will take steps to ensure that those with intellectual or developmental disabilities (IDD) and those with other nonphysical forms of disability are also included. As part of this effort, we have developed a working everyday language (the term preferred by members of the IDD community currently on our advisory committee, instead of “plain language”) definition of structural ableism (Textbox 1), and note this definition is expected to evolve.

**Figure 1:**
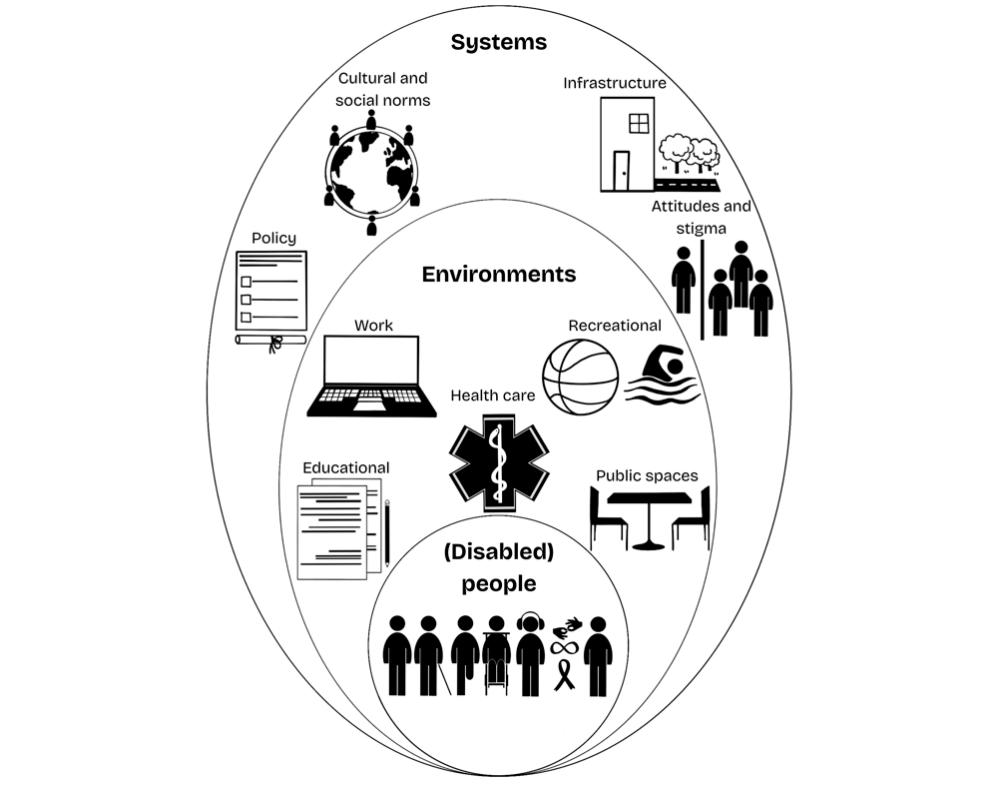
Early Visualization of Structural Ableism.

**Figure 2:**
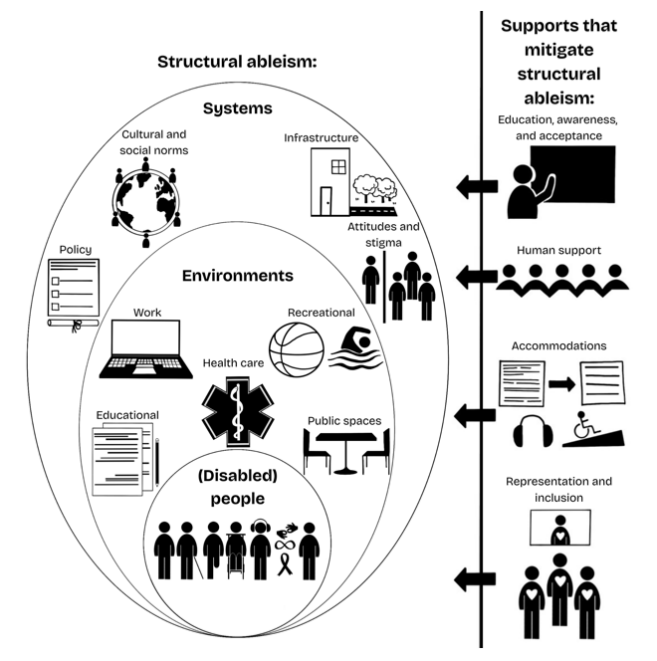
Early Visualization of Structural Ableism with Supports that Mitigate It.

The definition is accompanied by 2 graphics (Figures 1 and 2), which both represent our early understanding of structural ableism, as well as an early attempt to visualize structural ableism in a way that is accessible to those who use everyday language.

**Working everyday language definition of structural ableism and examples.**

Definition: Structural ableism means that the world is not designed to work for disabled people. It means that disabled people face more challenges than people without disabilities. For example, many places could be more accessible for people with disabilities, and many laws could better support disabled people. Disabled people are usually not asked about their opinions when decisions that matter to them are made. This means things stay unfair for them, and it is harder for them to live the life they want. Here are some examples:

- Workplace:
  Some jobs have policies that require many hours of work or don’t let employees work from home. This makes it hard for employees with energy-related disabilities to succeed.
- Education:
  Some schools lack the resources needed to provide students with audiobooks or handouts in large print. This makes it harder for some students with vision-related disabilities to learn. 
- Public spaces:
  Some public places, like restaurants and museums, were not built with a consideration for accessibility, so they lack ramps or elevators. This makes it difficult for people with mobility-related disabilities to enter them. 
- Recreational spaces:
  Not all recreational spaces have adequate social support because many people are not taught the importance of community support for the success and well-being of people with intellectual disabilities. This makes it harder for individuals with intellectual disabilities to participate in activities like sports.
- Health care:
  Most speech-language pathologists have their job because they want to help people. Sometimes, without realizing it, they help someone communicate in the way they think is best instead of in a way that is most helpful for that person, because they are not trained on how to best center the patient’s thoughts and opinions about their own care.
- Government policy:
  Medicaid was not made to consider all the needs of disabled people, and sets a very low savings limit. If disabled people save more than this, they lose coverage. This rule keeps people poor and stops them from saving for emergencies.

Learning from work about other forms of structural oppression [27-43], we understand that structural oppression is not limited to individual biases, and over time takes increasingly covert forms in social institutions while maintaining and reifying power imbalances [14,44]. For example, African American students with emotional, developmental, or learning disabilities are more likely than white students with the same disabilities to be suspended from school [45]. Such disparities deepen when considering variations in support needs and disability. This is demonstrated by people with autism spectrum disorder being less likely to receive vocational support than people with other disabilities [46]. We also note that, in this paper, we will be using the term oppression to express the experience of widespread unfair treatment, as it is the term used in other academic spaces. However, we acknowledge that, as identity and preference vary, this term may not resonate with all those in the disability community.

In addition to operating in conjunction with intersecting oppressed identities, structural ableism acts at both the individual and community levels. Examples at the individual level include: a patient who is deaf and not provided an American Sign Language (ASL) interpreter, a grandmother who uses a wheelchair and cannot visit her family because the building they live in does not have ramps, a college graduate with autism who cannot find a job due to employer biases, and a child with food allergies who struggles to find food options that are affordable and safe. The policies that uphold structural ableism create structural disparities that impact the disability community in all aspects of life: national shortages of ASL interpreters in hospital settings [47], higher rates of loneliness among older adults with disabilities than those without disabilities [48], higher unemployment rates among adults with autism [49], and an elevated percentage of children with food allergies who experience food insecurity [50]. Despite the knowledge that individuals with disabilities experience disparities that impact their health, there is still a dearth of information about the extent of these impacts.

As defined by the Americans with Disabilities Act (ADA), disability can be described as the experience of “a person who has a physical or mental impairment that substantially limits one or more major life activities, a person who has a history or record of such an impairment, or a person who is perceived by others as having such an impairment” [51]. This definition encompasses a variety of experiences, including those with mental health–related, intellectual, developmental, cognitive, sensory, and physical disabilities. Disability can be acquired at any point during one’s life, or be present since birth, can be both apparent or nonapparent to others [52], and varies depending on whether individuals are accommodated or not. Based on their environment and personal experiences, individuals may or may not identify as having a disability [53]. Structural ableism impacts all individuals with disabilities regardless of disability type or identity.

Measuring structural ableism is essential to creating interventions to slow and prevent it, ultimately improving health outcomes for disabled people by addressing the systems that create barriers to equitable health outcomes. Health inequities that impact disabled people are too often attributed to disabled people themselves and not the environment and contexts in which they live [54]. Yet, the experience of disability is multidimensional and contextual: it is social, cultural, and political, and is shaped by interactions with the environment [55-57]. Recent studies have shown the fallacy of attributing poor health outcomes solely to a disabled individual’s underlying health condition, demonstrating instead that ableism still permeates society, including in education [58], policing [59], employment [60], health care [61], and many other domains. Structural ableism is embedded ableism that drives poor health outcomes for the disability community. Consequently, health outcomes for disabled people can only be improved through a structural approach. Therefore, an essential first step toward addressing the health inequities that impact disabled people is to create comprehensive measures of structural ableism [13,14]. Clear measurement of structural ableism transforms invisible problems into visible ones. Such measures allow us to identify where further work is needed and the degree to which interventions have resulted in successes or deepened divides. It is only when we have ways to measure structural ableism that research can consider and address its effects on health outcomes [40].

Measurement of structural ableism must focus on all relevant structures with which disabled people interact. If measures are only inclusive of some aspects of the broader experience of structural ableism (eg, those that focus only on employment or education), they will fail to capture the full system contributing to negative health outcomes for the disability community [62]. Efforts to measure the multitude of domains relevant to structural racism, structural sexism, structural ageism, and other forms of structural oppression have identified relevant structures (eg, policies, leadership demographics, and hierarchies) spanning housing, education, criminal justice, employment, health care, political participation, immigration, and others [28,41,63]. Many similar domains are likely to be relevant to structural ableism, but the ways in which these structures operate to create injustice for disabled people may be unique [14]. Moreover, additional domains such as dimensions of the built environment [14,20,64] (eg, curb cuts) and dimensions of communication structures, often not highlighted in other measures of structural oppression, may have particular relevance for measuring structural ableism.

In addition to considerations of multiple domains, there is also a need to consider multiple levels. As other measures of structural oppression, including Patricia Homan’s work on structural sexism [40] and Adkins-Jackon et al [29] work on structural racism, account for both individual and community-level effects, measures of structural ableism must do the same [62]. Therefore, this work will focus on understanding and measuring the multidimensional and multilevel nature of structural ableism. This project will build on RSV’s and BKS’s *New England Journal of Medicine* paper, which outlined the need for measuring structural ableism [14], the team’s lived experiences as disabled people, and previous research efforts related to barriers for the disability community, and established expertise in all proposed methods.

We describe our methodological approach to developing measures of structural ableism. Our work lays the foundation for creating intersectional measures of structural oppression that are inclusive of structural ableism. Moving toward a structural intersectionality approach is critical, as dismantling structural oppression for all people will require understanding the ways multiple forms of structural oppression, including structural ableism, interact to shape health outcomes for people belonging to multiple health disparity populations [60,65,66]. In the future, we aim to build off the work of this project in order to create intersectional approaches to addressing structural ableism. The goal is to promote better health outcomes for all disabled and oppressed people, rather than promote the reinforcement of normativity.

## Methods

### Overview and Objectives

This project has 3 phases. In the first phase of this work, we will use historical, policy, and qualitative approaches to characterize the dimensions of structural ableism. The second phase develops and validates a survey-based individual-level measure of structural ableism. The third phase develops a community-level measure of structural ableism using publicly available datasets. This study has been approved by the University of Virginia (UVA) Institutional Review Board (IRB) for the Social and Behavioral Sciences. For all phases of the project, informed consent will be obtained, and participants will be compensated for their participation.

Our approach recognizes that addressing structural ableism must center the disability community and involve people living with a wide range of disabilities [13,14]. Our work achieves this at 3 levels. First, we will engage the disability community through an advisory committee comprised of community members. Second, our research team is dominated by members of the disability community. Third, we will engage participants in our research who are disabled. We will seek to have equal representation across all broad disability types: mobility and/or physical, cognitive, intellectual, and/or developmental, deaf and hard of hearing, communication disability, bodily difference, blind and visually impaired, mental health and psychological, and chronic illness [67,68]. More information on how we plan to engage with our advisory committee and research participants can be found in Table 1. We will prioritize accessibility in all aspects of this project. This includes ensuring all recruitment materials, data collection, tools, and deliverables are in plain language, 508 compliant, and Web Content Accessibility Guidelines (WCAG) 2.2 compliant [69-71]; 508 compliance indicates that all content complies with the accessibility standards for information and communication technology set by the United States Access Board, while WCAG 2.2 compliance indicates that materials comply with the international standard for accessibility of web content. We will additionally work with all participants to ensure accessibility needs are met. In order to meet the accessibility needs of each participant, interview questions are pasted into the chat on Zoom, which is particularly helpful for those who are deaf or hard of hearing or who have a disability related to language processing. A trained individual who is fluent in ASL leads interviews with participants who communicate with ASL. At times, there is also a team member speaking the interview questions so that closed captions are enabled and accessible during the interview. A trained individual with IDD leads interviews with participants who have IDD. Additionally, interview guides are provided to all participants ahead of time, and ample breaks are allowed throughout the process for those who need them. We anticipate that these methods will continue to be revised and expanded to meet the needs of our diverse participant population.

**Table 1:** Overview of disability community engagement.

Advisory committee	Research participants	Corresponding project phase
Advice on sourcing for historical and policy approach; review key informant interview questions	Informant interviews (n=30); targeted interviews and intake survey to select interviewees for diversity	Phase I
Mapping and review of the conceptual framework; review draft and validated individual-level measure of structural ableism	Semistructured cognitive interviews via Zoom (n=12); self-administered online survey (n=400+)	Phase II
Guide development of the conceptual framework; provide feedback on domains and indicators for community-level measures of structural ableism	Community engagement studio of disability advocates and activists (n=16); participating in the iterative design of a community-level measure of structural ableism	Phase III

### Study Design

#### Phase I: Characterizing the Multiple Domains That Comprise the Construct of Structural Ableism

In Phase I, we aim to characterize structural ableism, offering a broad overview of the dimensions of structural ableism and helping us identify these elements within a societal context. At the conclusion of Phase I, we will have developed a final overarching codebook that represents the dimensions of structural ableism and potential links between dimensions of structural ableism and health outcomes.

To understand how structural ableism is experienced by disabled people, it is important to listen to what disabled people have to share, to give them epistemic authority. Too often, disabled voices and perspectives are not centered in research work that impacts disabled people. To guide our examination of contemporary domains of structural ableism, we analyzed a variety of life writings by disabled authors and editors, along with policy documents and sources on the social and historical context of disability. We began with a commitment to a minimum of 50 sources as a matter of feasibility, limiting sources mostly to the last 20 years, but we have gone beyond this quota. We selected our sources for a diversity of types of text, authorship, and disability. We aimed to read widely to consider how the type of disability, race, gender, sexuality, and other aspects, such as geography and class, influenced experiences of ableism.

This approach examines historical events, ideologies, and the lived experiences of disabled people to understand the production and development of disability as a category and structural ableism as an oppressive force. We adopt a dual approach combining this historical perspective [44] with a policy perspective [34,55]. Recognizing that structural ableism is intricately linked to legal frameworks, our analysis further extends to both disability laws and to broader policies shaping the lives of disabled people [72,73]. This approach entails examining how laws and policies are written and interpreted, influencing the understanding and processing of disability, and helps us identify the dimensions of structural ableism from a policy standpoint. This information will inform the development of a summary of the material, identifying emergent themes and categories related to the dimensions of structural ableism and their relationships to health.

A qualitative approach will then be used to further characterize the dimensions of structural ableism [74,75]. We will engage 30 advocates and activists (or more as needed to reach saturation) from the disability rights and disability justice movements in key informant interviews which will combine aspects of oral history interviews and interviews that rely on the critical incident technique [76-78], thus combining approaches that allow us to understand experiences across the life course and also understand particularly memorable experiences.

Recruitment for participation in key informant interviews will begin by electronic distribution [69] of a brief survey to advocates. We will ask all who receive the survey to then send it on to other advocates and activists in the disability rights and disability justice movements with whom they are personally connected, and then ask those individuals to do the same. Our message accompanying this interest survey will contain the purpose of this study, a brief description of the purpose of the key informant interviews, eligibility criteria, and a note that not all who complete the survey will be invited to participate given our goal of including as diverse a group as possible in terms of disability type, advocacy type, gender identity, geographical location, race and ethnicity, and income status (maximum variance sampling). We will also share recruitment details via phone and in-person interactions to let advocates and activists in the disability community who may not be online know of the opportunity to participate. To be eligible, individuals must identify as an advocate or activist in the disability rights or disability justice movements, be 18 years of age or older, and live within the United States. They must further identify as disabled or have a close, personal relationship with a disabled individual (eg, caregiver or parent).

We expect each interview to last for approximately 90 minutes, with more time provided as needed. During the interviews, we will use a semistructured interview guide to explore how the individuals themselves and members of the disability community more broadly experience frustrations, barriers, and exclusion. The interview guides used in this study are available in Multimedia Appendices 1-5. We will begin by asking broad questions about these types of experiences across the life course, and then probe into experiences related to specific aspects of life, including education, employment, community spaces, transportation, criminal justice, and housing.

We will use an inductive approach to qualitative content analysis, in which themes and categories are inductively and iteratively derived from the data [79]. Our analysis will begin with investigators independently coding 3 transcripts from the qualitative interviews and then coming together to create an initial codebook through a consensus-building process. The remaining transcripts will then be divided among 4 research assistants for coding. All coding will take place within QSR NVivo.

At the completion of the historical and policy review and the key informant interviews, we will develop an overarching codebook that represents the dimensions of structural ableism and ways in which our 2 types of sources discuss links between dimensions of structural ableism and health outcomes. To establish rigor in our interpretive work, we will use source and analyst triangulation; peer debriefing; reflexive journaling; the presentation of quotes, both from texts and transcripts; and we will make data available upon request [80,81].

#### Phase II: Develop and Validate an Individual-Level Measure of Structural Ableism

In the second phase, we will qualitatively pretest and quantitatively assess the validity of an original survey instrument that will serve as an individual-level measure of structural ableism. This instrument will be grounded in Phase 1 findings and will provide a way to measure the relationship between individual experiences with structural ableism and health outcomes.

First, we will develop a detailed conceptual framework that identifies the various dimensions and corresponding specific items within each dimension of structural ableism that the survey questionnaire is intended to capture. All items will be developed in plain language. This model will also include the eligibility criteria and respondent characteristics to be measured, such as geographic location, age, gender, race and ethnicity, and socioeconomic background.

The research team will conduct a series of cognitive interviews to pretest the survey instrument with disabled individuals ahead of pilot survey administration [82-84]. This study proposes to conduct 12 interviews, or more as needed to reach saturation, to evaluate the instrument for clarity, accessibility, and face validity. Cognitive interviews are expected to be approximately 60 minutes. During the interview, the facilitator will verbally and/or visually review each question of the survey instrument. We will ask participants to articulate their interpretation of specific concepts and terms included in the survey, to rephrase the questions in their own words, to explain the thought processes they followed to come up with the answer that they did, and to explain their rationales for the responses they provided. Additionally, through the use of probing questions, the facilitator may ask the participant for additional clarification or explanation. Recruitment and sampling for the cognitive interview will parallel that described for the key informant interview in Phase I.

After the completion of each interview, the facilitator will synthesize the interview feedback into a detailed narrative summary report, identifying any questions or answers that posed challenges for the participant. Separately, a facilitator who did not participate in the interview will review the recording from the interview and prepare their own summary report of the feedback. At the conclusion of the interview data collection period, we will review all interview summaries and prepare a synthesized report of findings across interviews and the resulting recommendations for revisions to the survey instrument. The resulting report of recommendations will be shared with cognitive interview participants for member checking and will be revised in response to the feedback received. We will revise the survey questions and answer categories as needed based on the pretest interview feedback. We will then pilot test the revised instrument through online survey administration to determine usability.

Next, we will conduct pilot survey administration and validate measures through psychometric analyses. The pilot survey administration and recruitment will occur online and draw from multiple sampling strategies. The survey will be hosted by the UVA’s Center for Survey Research on the University’s license of Qualtrics. Currently, 15 question types within Qualtrics are WCAG 2.0 AA compliant.

All participants for the survey pilot will be adults living in the United States who have one or more disabilities. Eligibility will be based on the participant either self-identifying as having a disability and/or chronic illness or self-identifying as having a functional limitation. Screening questions for eligibility will include the following: (1) Do you have a disability and/or chronic illness? (2) What types of disabilities do you have? Select all that apply. (3) Six questions from the American Community Survey, which inquire about difficulties across different domains of functioning (difficulties hearing, seeing, concentrating, or making decisions, walking or climbing stairs, dressing or bathing, and doing errands alone) [83]. An individual will be eligible if they self-select as identifying with at least one of these screening items. These screening measures include functional limitations as well as self-identifying as having a disability or chronic illness (and given the space to expand on that identification) in an effort to be comprehensive, recognizing that relying on just one or the other could result in a failure to capture the full range of respondents with relevant experiences. Eligible individuals will be 18 years of age or older. Given the specific eligibility criteria, nonprobability sampling methods are best suited to efficiently reach this population for the pilot. The self-administered survey is expected to take 20-30 minutes to complete, although we expect that time will vary across the disability community. The survey will be administered in English. We will offer survey support throughout data collection and provide both an email and phone number for participants to contact should they have questions or need assistance with or accommodations for completing the online questionnaire.

We will use two sources for survey participants: (1) an online access panel (Dynata [85], which includes over 70 million potential respondents and has a commitment to broad representation in their panelists across multiple identities including disability and chronic illness) and (2) indirect recruitment through the listservs and social media accounts of the project team and advisory committee’s expansive network of disabled people, organizations and researchers. From the online panel, we will collect 400 responses. Due to the nature of the indirect recruitment through listservs and social media, it is unknown how many additional responses we may receive through these channels. The count of 400 achieved through the online panel will ensure we have the minimum number of completions needed to support the range of analyses planned to validate the instrument.

To assess the reliability and validity of the survey scale, we will conduct a series of psychometric analyses using pilot survey data, which will be prepared and analyzed in SPSS (IBM Corp). We will first calculate Cronbach ⍺ to assess the reliability of the scale, specifically the internal consistency of the items measuring structural ableism. To investigate possible item reduction, we will run inter-item correlations and adjusted item-total correlations. Given that this is a newly developed scale, we will conduct exploratory factor analysis to assess the underlying factor structure within the data, followed by confirmatory factor analysis. To further assess construct validity, we will use correlation analysis, calculating the Pearson correlation coefficient (r) between the structural ableism measures and both theoretically related and unrelated measures to test for convergent validity and discriminant validity, respectively. As an additional assessment of construct validity, we will run a series of bivariate and multivariate regressions between the scale values and relevant health outcomes. If any of these analyses lead to substantial changes, we will administer a second, revised questionnaire, piloting the survey to a new, independent sample of 400 respondents from the online survey panel, controlling for the exclusion of any respondents who had participated in the prior survey. We will then rerun all analyses to test the psychometric properties of the revised instrument. These methods of psychometric analysis are in alignment with best practices for scale development [86].

Our survey will include 4 questions related to health outcomes: (1) having a usual health care provider, (2) not having any health insurance, (3) having an unmet health need due to cost, and (4) having a routine check-up in the past 12 months [87]. These outcome measures were selected because previous research has demonstrated health disparities between disabled and nondisabled people [88-90], and they are commonly used metrics across the public health and health care fields when creating medical surveys. To explore these relationships, we will run a series of nested model regressions of structural ableism across the 4 health outcomes of our individual-level measure of structural ableism. These analyses will test our hypothesis that higher levels of individual-level structural ableism are associated with greater barriers across these 4 indicators.

Phase II will result in a validated instrument to quantitatively measure individuals’ experiences with structural ableism and will explore the relationship between this measure of structural ableism and health outcomes.

#### Phase III: Measure Structural Ableism at a Community Level Using Publicly Available Datasets

Phase III aims to create a community-level measure of structural ableism in order to explore its relationship with health outcomes.

We will use an iterative design approach to select potential indicators for the composite measure of structural ableism. This process will be informed by qualitative data collected from Phase I and feedback from our advisory committee and will help us outline content domains and potential indicators relevant to structural ableism at a community level (eg, food security, transportation, employment, etc). The resulting composite measure will be revised with advocates and activists in the disability rights and disability justice movements through 2 community engagement studios [91,92], an established methodology that facilitates bidirectional input between the research team and the disability community via virtual gatherings and facilitated discussion [93]. This approach ensures that the lived experiences of people with disabilities are prioritized in the selection of indicators and item development.

### Community Engagement Studios

Sixteen advocates and activists in the disability rights and disability justice movements will participate in the community engagement studios. Eligible individuals will be 18 years of age or older, self-identify as an advocate or activist in the disability rights or disability justice movements, and live in the United States. We expect that about half of those engaged in the community engagement studios will have participated in the Phase I qualitative interviews.

Recruitment for participation in the community engagement studios will parallel the recruitment process described for the Phase I key informant interviews. We will host 2 community engagement studios: one at the start to gain further insight on the indicators of structural ableism identified in Phase I, and a second studio to include community feedback in the final selection of metrics. During each studio, we will pose 3 to 4 questions to obtain feedback on the range of dimensions of structural ableism represented, the particular datasets and questions to be used, and any additional overarching feedback. The community engagement studio sessions will last approximately 90 minutes, and material for discussion will be distributed beforehand in formats accessible to each participant.

After completion, the facilitator will synthesize the feedback into a detailed narrative summary report, identifying all points of feedback from the participants. Separately, another member of the research team who did not participate in the community engagement studio will listen to the audio recording and prepare their own summary report of the feedback. The consensus summary will be shared with community engagement studio participants for member checking and will be revised accordingly.

### Development of Indicators

To develop indicators of structural ableism, we will use data primarily from the Behavioral Risk Factor Surveillance System, a state-based national survey of noninstitutionalized adults 18 years and older, to investigate the same health outcome measures stated in Phase II [87]. As a secondary step, we will explore linking indicators to data from the IPUMS Health Surveys, which are a comprehensive integration of health survey data from sources such as the National Health Interview Survey [94]. These datasets assess disability status using the American Community Survey Questions on Disability (ACS-6), assessing difficulties across different domains of functioning (difficulties hearing, seeing, concentrating or making decisions, walking or climbing stairs, dressing or bathing, and doing errands alone). This process will include developing a menu of indicators of structural ableism based on the themes emerging from Phase I and supplemented by input from our advisory committee and a review of pertinent literature. We will then examine existing variables available in the Behavioral Risk Factor Surveillance System, and available indicators will move forward to the next steps of the process.

Since our analysis is mostly focused on capturing gaps between disabled and nondisabled people, we propose to use the approach used by Dougherty et al [95]. A composite measure of structural ableism will be estimated using confirmatory factor analysis, which minimizes the effect of random measurement error by accounting for the variability across prespecified indicators in a summary factor. Unidimensional factor models will be produced using the composite measure as the single latent variable with a mean of 0 and SD of 1. Robust maximum likelihood estimation will be used to produce unbiased estimates, and model fit will be evaluated using a Tucker-Lewis index. Candidate models would at least contain one indicator per content domain, and a final model will be chosen, assessing the trade-off between model complexity and goodness of fit [96].

For each indicator, prevalence ratios will be computed, log-transformed, and normalized using robust min-max scaling relying on prespecified percentiles (2.5th and 97.5th). Values outside of that percentile range will be winsorized to those bounds, and the resulting bounded values will be linearly transformed to a 0-100 scale.

Phase III will result in the development of novel indicators of structural ableism identified by disabled people available from nationally representative surveys, and the development of a composite measure of structural ableism based on these measures.

### Ethical Considerations

All aspects of this study will be conducted in a manner that is HIPAA (Health Insurance Portability and Accountability Act)-compliant and accountable under the responsible conduct of research protocols active at the UVA. A Human Subjects application has been approved by UVA’s IRB for the Social and Behavioral Sciences (protocol number 6677), which operates in accordance with the Belmont Report, the Nuremberg Code, and the Declaration of Helsinki. The UVA IRB for the Social and Behavioral Sciences will serve as the single IRB for this study. This research falls under the classification of nonexempt human participants’ research. The privacy of all participants will be maintained through the deidentification of all identifiable data, which will be stored on a secure server. Participant data will be linked to a participant ID number. All participants will receive a consent document in plain language, which will be kept on a secure server at UVA after being signed. For engagement in key informant interviews and community engagement studios, all participants will receive US $150 in compensation. For participation in the cognitive interviews, all participants will receive US $100 in compensation. No compensation will be provided directly to survey participants for Phase 2.

No prisoners or institutionalized individuals will be involved in the proposed research. We recognize that, as 66% of incarcerated individuals report having a disability [13], approximately 3% of disabled individuals live in institutional group quarters [97], and people with disabilities are more likely to experience state-sanctioned violence [13], excluding incarcerated and institutionalized individuals creates limitations on capturing the full experience of structural ableism. However, we will include historical narratives that explore the experience of incarceration and institutionalization, and will work to include disabled individuals who have previously experienced both incarceration and institutionalization.

## Results

This project was funded in June of 2024. As of October 2025, our team has read over 50 texts as part of our historical and policy analysis of the factors that characterize structural ableism. We plan to complete our characterization of structural ableism in the spring of 2026, with individual-level measures of structural ableism being developed by the Winter of 2028 and community-level measures created by the Winter of 2029.

## Discussion

### Anticipated Findings

The disability community is in urgent need of systems-based solutions to improve health equity [14]. This project lays the essential groundwork for understanding the etiology of these disparities and the pathways that must be disrupted to achieve health equity for people with disabilities. We anticipate that the findings of our project will result in a multidimensional characterization of structural ableism that includes both individual and community-level measures. The Phase I deliverable of characterizing the factors that comprise structural ableism will be validated through analysis of texts and key informant interviews that will aim to capture the diverse ways individuals can be impacted by structural ableism. The Phase II deliverable of developing an individual-level measure of structural ableism will be validated through surveys conducted with members of the disability community. Finally, the Phase III deliverable of developing a community-level measure of structural ableism will be validated through the use of publicly available datasets. We hypothesize that the measures developed upon the completion of the 3 phases of this project will inform a new understanding of the relationship between health disparities and structural ableism in order to improve health outcomes of the disability community.

Across all 3 phases, we take an intersectional, interdisciplinary, and community-grounded approach [65]. We purposefully include disabled people across all steps and phases of this project, with a focus on maximizing diversity of disability perspectives by including people across disability types and with intersectional identities (eg, race and ethnicity, gender identity, and geographic location) [60,67,98]. Our interdisciplinary team has expertise in disability studies, public health, medicine, health policy, systems engineering, sociology, and cultural anthropology, and is led by 2 principal investigators with disabilities (RSV and BKS). Our approach is deeply community-informed, drawing on multiple community partnerships from local and national organizations in the context of our advisory committee and drawing on engagement directly with the disability community across all 3 aims and dissemination activities.

As we begin this project, we have made multiple decision points, primarily pertaining to how this work can be made as inclusive as possible. First, we have been working with the disability community to determine the best ways to have our work resonate with those who live with functional limitations or chronic illness but do not identify as part of the disability community. We want the measures we develop to reflect all who experience the barriers caused by disability, not just those who identify as disabled. Second, as pertaining to dissemination, we have made considerations around balancing ways to make the study as inclusive as possible with the funding and resources we have available. While we are working within these constraints, we are also searching for opportunities for additional funding to expand in directions that will make our work more inclusive and accessible. This includes a project funded by the WITH foundation in 2025 that focuses on using artificial intelligence to create plain language materials. Representing our work in plain language, incorporating artwork to visualize the concepts discussed, and finding ways to include the experiences of deaf individuals and ASL users are all areas being evaluated to improve dissemination and accessibility.

### Conclusions

The measures developed by this work will create a foundation for identifying and evaluating novel interventions aimed at dismantling structural ableism. These interventions should be co-created with the disability community.

## Appendix

Multimedia Appendix 1 Spanish language identify first Phase I interview guide.

Multimedia Appendix 2 Spanish language person first Phase I interview guide.

Multimedia Appendix 3 English language Phase I identify first interview guide.

Multimedia Appendix 4 English language Phase I person first interview guide.

Multimedia Appendix 5 Link to ASL Phase I interview guide video.

## Abbreviations

ACS-6: American Community Survey Questions on Disability
ADA: Americans With Disabilities Act
ASL: American Sign Language
HIPAA: Health Insurance Portability and Accountability Act
IDD: intellectual and developmental disabilities
IRB: Institutional Review Board
UVA: University of Virginia
WCAG: Web Content Accessibility Guidelines

## Data Availability

Data sharing is not applicable to this article as no data have yet been generated from the study.
