# Supplementary material for: Developing and Validating Measures of Structural Ableism to Improve Health Outcomes for the Disability Community: Protocol for a Mixed Methods Study
Source: JMIR Res Protoc. 2026 Mar 13;15:e86976. doi: 10.2196/86976 (PMC13032091; doi:10.2196/86976)
Supplement: Multimedia Appendix 1 [file resprot_v15i1e86976_app1.docx]

**Guía para Entrevistas Semiestructuradas**

1. Piense en una ocasión en la que haya sido tratado injustamente debido a su discapacidad. ¿Qué es lo primero que le viene a la mente?
2. Piense en los retos o frustraciones a los que se enfrentan las personas con discapacidad. ¿Qué es lo primero que le viene a la mente?
3. ¿Por qué cree que las personas con discapacidad no reciben el mismo trato que las personas sin discapacidad?
   1. *Pregunta: ¿De qué manera cree que las personas con discapacidad no reciben el mismo trato que las personas sin discapacidad?*
4. ¿En qué se diferencian los retos o frustraciones de las personas con discapacidad desde pequeños y los de las personas que adquieren una discapacidad en la edad adulta?
5. ¿Cómo cree que otras identidades y experiencias influyen en la experiencia de una persona con discapacidad?

***Pensemos en diferentes aspectos de la vida. Empecemos por la escuela.***

1. Piense en los retos a los que se ha enfrentado personalmente en la escuela debido a su discapacidad o discapacidades. ¿Qué es lo primero que le viene a la mente?
2. Piense en los retos o frustraciones a los que se enfrentan las personas con discapacidad en la escuela. ¿Qué es lo primero que le viene a la mente?
3. ¿En qué se diferencian los retos o frustraciones en la escuela de las personas con discapacidad desde una edad temprana y las de las personas que adquieren una discapacidad en la edad adulta?

*Preguntas*

- 1. *¿Qué tipo de retos o frustraciones enfrentan las personas con discapacidad cuando solicitan adaptaciones en la escuela?*
  2. *¿Qué tipo de retos o frustraciones enfrentan las personas con discapacidad con sus amigos o profesores en la escuela?*

***Ahora pensemos en el trabajo.***

1. Piense en los retos o frustraciones que ha enfrentado personalmente en el trabajo debido a su discapacidad o discapacidades. ¿Qué es lo primero que le viene a la mente?
2. Piense en los retos o frustraciones a los que se enfrentan las personas con discapacidad en el trabajo. ¿Qué es lo primero que le viene a la mente?
3. ¿En qué se diferencian los retos o frustraciones en el trabajo de las personas con discapacidad desde pequeños y de las que lo son desde mayores?

*Preguntas*

- 1. *¿Qué tipo de retos o frustraciones enfrentan las personas con discapacidad cuando intentan encontrar un trabajo que les satisfaga?*
  2. *¿Qué tipo de retos o frustraciones enfrentan las personas con discapacidad cuando solicitan adaptaciones en el trabajo?*
  3. *¿Qué tipo de retos o frustraciones enfrentan las personas con discapacidad en sus relaciones con sus compañeros de trabajo?*

***Ahora pensemos en el lugar donde vive la gente.***

1. Piense en los retos o frustraciones que ha enfrentado personalmente en su hogar o en su comunidad debido a su discapacidad o discapacidades. ¿Qué es lo primero que le viene a la mente?
2. Piense en los retos o frustraciones a los que se enfrentan las personas con discapacidad en sus hogares y comunidades. ¿Qué es lo primero que le viene a la mente?
3. ¿Por qué cree que a las personas con discapacidad les cuesta tanto encontrar hogares y comunidades que sean totalmente accesibles para ellas?
4. ¿En qué se diferencian los retos o frustraciones en los hogares y comunidades de las personas con discapacidad desde pequeños y los de las personas que adquieren una discapacidad en la edad adulta?

*Preguntas*

- 1. *¿Qué tipo de retos o frustraciones enfrentan las personas con discapacidad cuando intentan hacer amigos en sus comunidades?*
  2. *¿Qué tipo de retos o frustraciones enfrentan las personas con discapacidad cuando se desplazan (por ejemplo, por las aceras) o utilizan los espacios públicos de sus comunidades?*

***Ahora pensemos en cómo llegas a los sitios.***

1. Piense en los retos o frustraciones que ha enfrentado personalmente debido a su discapacidad o discapacidades al intentar llegar a algún lugar. ¿Qué es lo primero que le viene a la mente?
2. Piense en los retos o frustraciones a los que se enfrentan las personas con discapacidad cuando intentan llegar a algún sitio. ¿Qué es lo primero que le viene a la mente?
3. ¿Por qué cree que las personas con discapacidad tienen dificultades para encontrar un medio de transporte que sea totalmente accesible para ellas?
4. ¿En qué se diferencia la experiencia con el transporte de las personas con discapacidad que viven en pueblos pequeños de la de las que viven en ciudades?

*Preguntas*

- 1. *¿Qué tipo de frustraciones o retos afrontan las personas con discapacidad al utilizar el transporte público?*
  2. *¿Qué tipo de frustraciones o dificultades enfrentan las personas con discapacidad al conducir o utilizar un vehículo personal?*
  3. *¿Qué tipo de frustraciones o retos afronta una persona con discapacidad si no conduce un vehículo?*
  4. *¿Qué tipo de frustraciones o dificultades enfrentan las personas con discapacidad al utilizar servicios de transporte compartido como Uber o Lyft?*

***Ahora pensemos en ir al médico.***

1. Piense en los retos o frustraciones que has enfrentado personalmente debido a su discapacidad o discapacidades al ir al médico. ¿Qué es lo primero que le viene a la mente?
2. Piense en los retos o frustraciones a los que se enfrentan las personas con discapacidad cuando van al médico. ¿Qué es lo primero que le viene a la mente?
3. ¿En qué se diferencia esta experiencia para las personas con diferentes tipos de discapacidad?

*Preguntas*

- 1. *¿Qué tipo de frustraciones o retos afrontan las personas con discapacidad cuando necesitan atención médica de urgencia?*
  2. *¿Qué tipo de retos o frustraciones enfrentan las personas con discapacidad al pagar la atención que necesitan?*
  3. *¿Qué tipo de retos o frustraciones enfrentan las personas con discapacidad cuando acuden a centros de salud físicos, como hospitales o clínicas?*

*Ahora podemos tomar un breve descanso, si lo desean.*

*«Antes de continuar, quiero preguntarle: ¿le parece bien seguir con la entrevista?».*

Si la respuesta es NO, pregunte un poco por qué y pida cambiar la cita para otro momento.

Si la respuesta es SÍ, continúe.

***Ahora pensemos en lugares a los que la gente va a jugar y divertirse (por ejemplo, un parque, una piscina, una sala de juegos, un gimnasio, un cine, etc.).***

1. Piense en los retos o frustraciones que has enfrentado personalmente debido a su discapacidad cuando va a algún lugar para jugar y divertirse. ¿Qué es lo primero que le viene a la mente?
2. Piense en los retos o frustraciones a los que se enfrentan las personas con discapacidad cuando van a algún lugar para jugar y divertirse. ¿Qué es lo primero que le viene a la mente?
3. ¿En qué se diferencian los retos o frustraciones de las personas con discapacidad desde pequeños y los de las personas que adquieren una discapacidad en la edad adulta?

***Ahora pensemos en la tecnología.***

1. Piense en los retos o frustraciones que usted mismo ha experimentado debido a su discapacidad al utilizar la tecnología. ¿Qué es lo primero que le viene a la mente?
2. Piense en los retos o frustraciones a los que se enfrentan las personas con discapacidad cuando utilizan la tecnología. ¿Qué es lo primero que le viene a la mente?
3. ¿En qué se diferencian los retos o frustraciones de las personas con discapacidad desde pequeños y los de quienes la adquieren en la edad adulta?

*Preguntas*

- 1. *¿Qué tipo de retos o frustraciones enfrentan las personas con discapacidad al utilizar aplicaciones o herramientas diseñadas específicamente para ayudar a las personas con discapacidad?*

***Ahora pensemos en cómo se representa la discapacidad en los medios de comunicación.***

1. Piense en cómo se representa la discapacidad en los medios de comunicación. ¿Qué retos o frustraciones enfrentan las personas con discapacidad para ser representadas adecuadamente en los medios de comunicación?
2. ¿Cómo cambia esta representación cuando las personas con discapacidad participan en la creación de contenidos mediáticos en comparación con cuando no participan?

***Ahora pensemos en la policía.***

1. Piense en los retos o frustraciones que ha enfrentado personalmente con la policía. ¿Qué es lo primero que le viene a la mente?
2. Piense en los retos o frustraciones que tienen las personas con discapacidad con la policía. ¿Qué es lo primero que le viene a la mente?
3. ¿En qué se diferencia esta experiencia para las personas con diferentes tipos de discapacidad?

*Preguntas*

- 1. *¿Qué tipo de problemas de seguridad pueden enfrentar las personas con discapacidad al tratar con la policía?*
  2. *¿Cómo cree que los agentes de policía entienden o responden a los diferentes tipos de discapacidad (por ejemplo, física, cognitiva, psiquiátrica)?*

***Ahora pensemos en mudarse a este país desde otro país***

1. ¿Tiene alguna experiencia personal sobre mudarte a este país desde otro país?
   1. [Si la respuesta es SÍ] ¿Cuáles son los retos o frustraciones a los que se ha enfrentado personalmente?
2. Piense en los retos o frustraciones a los que se enfrentan las personas con discapacidad que se han mudado a este país desde otro país. ¿Qué es lo primero que le viene a la mente?
3. ¿En qué se diferencia esta experiencia para las personas con discapacidad que son inmigrantes documentados de la de los inmigrantes indocumentados?

***Ahora pensemos en el voto.***

1. Piense en los retos o frustraciones que ha enfrentado personalmente al votar. ¿Qué es lo primero que le viene a la mente?
2. Piense en los retos o frustraciones a los que se enfrentan las personas con discapacidad cuando intentan votar. ¿Qué es lo primero que le viene a la mente?
3. ¿En qué se diferencia esta experiencia para las personas con discapacidad que viven en centros colectivos en comparación con las que viven en una comunidad?

*Preguntas*

- 1. *¿Qué tipo de retos o frustraciones enfrentan las personas con discapacidad cuando votan en persona (por ejemplo, acceso físico, tecnología, interacción con los trabajadores electorales)?*
  2. *¿Qué tipo de retos o frustraciones enfrentan las personas con discapacidad al votar mediante voto por correo?*

¿En qué otros aspectos de la vida cree que las personas con discapacidad experimentan retos o frustraciones?

*[Para cada ámbito que propongan los participantes, plantee las siguientes preguntas:]*

1. Piense en los retos o frustraciones a los que se enfrentan las personas con discapacidad cuando [inserte el ámbito mencionado por el participante]. ¿Qué es lo primero que le viene a la mente?
2. ¿De qué manera cree que las personas con discapacidad no reciben el mismo trato que las personas sin discapacidad en [inserte el ámbito mencionado por el participante]?
3. ¿En qué se diferencian los retos o frustraciones en [insertar el ámbito mencionado por el participante] para las personas con discapacidad desde pequeños y para las que lo son desde una edad más avanzada?
4. ¿Por qué cree que las personas con discapacidad no reciben el mismo trato que las personas sin discapacidad en [inserte el ámbito mencionado por el participante]?
5. ¿Cómo cree que otras identidades y experiencias dan forma a la experiencia de una persona con discapacidad en [insertar ámbito mencionado por el participante]?

**Técnica de incidentes críticos**

1. Cuénteme alguna ocasión en la que se sintió tratado injustamente por su discapacidad.
   1. ¿Puede contarme qué pasó?
   2. ¿Puede decirme cómo se sintió?
   3. ¿Puede decirme por qué cree que le trataron injustamente?
   4. ¿Qué le hubiera gustado que hubiera pasado en su lugar?
2. Cuénteme alguna ocasión en la que se sintió tratado con respeto y se atendieron todas sus necesidades relacionadas con su discapacidad.
   1. ¿Puede contarme qué pasó?
   2. ¿Puede decirme cómo se sintió?
   3. ¿Puede decirme por qué cree que le trataron con respeto?
3. ¿Puede hablar de alguna ocasión en la que alguien prefirió tratar con otra persona en lugar de tratar con usted directamente?
   1. ¿Puede contarme qué pasó?
   2. ¿Puede decirme cómo se sintió?
   3. ¿Puede decirme por qué cree que prefirieron tratar con otra persona en lugar de con usted?
   4. ¿Qué le hubiera gustado que hubiera pasado en su lugar?

*Gracias por tomarse el tiempo de hablar con nosotros hoy. Sus respuestas nos ayudarán a comprender cómo las personas con discapacidad sufren un trato injusto, para que podamos encontrar formas de garantizar que esto no ocurra con tanta frecuencia.*

*Al final del estudio, compartiremos nuestros resultados con la comunidad de personas con discapacidad y con otros investigadores. ¿Quiere que otras personas sepan que ha participado en este estudio?*

Si el participante responde SÍ:

*Podemos hacerlo de dos maneras:*

*1) Podemos utilizar su nombre junto a sus palabras.*

*2) Podemos hacer saber a la gente que usted formó parte de este estudio sin decir cuáles fueron sus palabras.*

Si el participante responde NO:

*Nos aseguraremos de que su nombre permanezca en el anonimato.*
