# Supplementary material for: Developing and Validating Measures of Structural Ableism to Improve Health Outcomes for the Disability Community: Protocol for a Mixed Methods Study
Source: JMIR Res Protoc. 2026 Mar 13;15:e86976. doi: 10.2196/86976 (PMC13032091; doi:10.2196/86976)
Supplement: Multimedia Appendix 3 [file resprot_v15i1e86976_app3.docx]

**Semi- Structured Interview Guide**

**Identity First Language**

1. Think about a time when you were treated unfairly because of your disability. What are the first things that come to mind?
2. Think about the challenges or frustrations faced by disabled people. What are the first things that come to mind?
3. Why do you think disabled people are not treated the same as non-disabled people?
   1. *Probe: In what ways do you think disabled people are not treated the same as non-disabled people?*
4. How are the challenges or frustrations different for those who are disabled from a young age than those who become disabled when they are older?
5. How do you think other identities and experiences shape a disabled person’s experience?

***Let’s think about different parts of life. Let’s start with school.***

1. Think about challenges you have personally faced at school because of your disability(ies)? What are the first things that come to mind?
2. Think about the challenges or frustrations faced by disabled people at school. What are the first things that come to mind?
3. How are the challenges or frustrations different at school for those who are disabled from a young age than those who become disabled when they are older?

*Probes*

- 1. *What kind of challenges or frustrations do disabled people face when asking for accommodations at school?*
  2. *What kind of challenges or frustrations do disabled people face with their friends or teachers at school?*

***Now let’s think about work.***

1. Think about challenges or frustrations you have personally faced at work because of your disability(ies). What are the first things that come to mind?
2. Think about the challenges or frustrations faced by disabled people at work. What are the first things that come to mind?
3. How are the challenges or frustrations different at work for those who are disabled from a young age than those who become disabled when they are older?

*Probes*

- 1. *What kind of challenges or frustrations do disabled people face when trying to find a job that feels right for them?*
  2. *What kind of challenges or frustrations do disabled people face when asking for accommodations at work?*
  3. *What kind of challenges or frustrations do disabled people face in their relationships with the people they work with?*

***Now let’s think about where people live.***

1. Think about challenges or frustrations you have personally faced in your home or your community because of your disability(ies)? What are the first things that come to mind?
2. Think about the challenges or frustrations disabled people face in their homes and communities. What are the first things that come to mind?
3. Why do you think disabled people have a hard time finding homes and communities that are fully accessible to them?
4. How are the challenges or frustrations different in homes and communities different for those who are disabled from a young age than those who become disabled when they are older?

*Probes*

- 1. *What kind of challenges or frustrations do disabled people face when trying to make friends in their communities?*
  2. *What kind of challenges or frustrations do disabled people face when moving around (e.g. sidewalks) or using public spaces in their communities?*

***Now let’s think about how you get to places.***

1. Think about challenges or frustrations you have personally faced because of your disability(ies) when trying to get somewhere. What are the first things that come to mind?
2. Think about the challenges or frustrations faced by disabled people when trying to get somewhere. What are the first things that come to mind?
3. Why do you think disabled people have a hard time finding transportation that is fully accessible to them?
4. How is the experience with transportation different for disabled people who live in small towns than those who live in cities?

*Probes*

- 1. *What kind of frustrations or challenges do disabled people face when using public transportation?*
  2. *What kind of frustrations or challenges do disabled people face when driving or using a personal vehicle?*
  3. *What kind of frustrations or challenges does someone disabled face if they do not drive a vehicle?*
  4. *What kind of frustrations or challenges do disabled people face when using ride sharing services like Uber or Lyft?*

***Now let’s think about going to see a doctor.***

1. Think about challenges or frustrations you have personally faced because of your disability(ies) when going to see a doctor. What are the first things that come to mind?
2. Think about the challenges or frustrations faced by disabled people when going to see a doctor. What are the first things that come to mind?
3. How is this experience different for those with different types of disabilities?

*Probes*

- 1. *What kind of frustrations or challenges do disabled people face when seeking emergency medical care?*
  2. *What kind of challenges or frustrations do disabled people face when paying for the care they need?*
  3. *What kind of challenges or frustrations do disabled people face when going to physical healthcare spaces like hospitals or clinics?*

***“We can now take a short break if you would like.”***

***“Before we begin again, I want to ask: are you okay with continuing this interview?”***

**If NO, ask for a reason and if the participant would like to reschedule for another time.**

**If YES, continue.**

***Now let’s think about places where people go to play and have fun (for example, a park, swimming pool, arcade, gym, movie theatre, etc.).***

1. Think about challenges or frustrations you have personally faced because of your disability(ies) when going somewhere to play and have fun. What are the first things that come to mind?
2. Think about the challenges or frustrations disabled people face when going somewhere to play and have fun. What are the first things that come to mind?
3. How are the challenges or frustrations different for those who are disabled from a young age than those who become disabled when they are older?

***Now let’s think about technology.***

1. Think about challenges or frustrations you have personally because of your disability(ies) when using technology. What are the first things that come to mind?
2. Think about the challenges or frustrations disabled people face when using technology. What are the first things that come to mind?
3. How are the challenges or frustrations different for those who are disabled from a young age than those who become disabled when they are older?

*Probes*

- 1. *What kind of challenges or frustrations are faced by disabled people when using applications or tools specifically designed to help people with disabilities?*

***Now let’s think about how disability is represented in the media.***

1. Think about how disability is represented in the media. What challenges or frustrations do disabled people face with being appropriately represented in the media?
2. How does this representation change when disabled people are involved in creating media content compared to when they are not involved?

***Now let’s think about the police.***

1. Think about challenges or frustrations you have personally faced with the police. What are the first things that come to mind?
2. Think about the challenges or frustrations disabled people have with the police. What are the first things that come to mind?
3. How is this experience different for those with different types of disabilities?

*Probes*

- 1. *What kinds of safety concerns might disabled people face when dealing with the police?*
  2. *How do you think police officers understand or respond to different kinds of disabilities (e.g., physical, cognitive, psychiatric)?*

***Now let’s think about moving to this country from another country***

1. Do you have any personal experience moving to this country from another country?
   1. [If YES] What are the challenges or frustrations that you have personally faced?
2. Think about the challenges or frustrations faced by disabled people who moved to this country from another country. What are the first things that come to mind?
3. How is this experience different for disabled people who are documented immigrants than those who may be undocumented immigrants?

***Now let’s think about voting.***

1. Think about challenges or frustrations you have personally faced when voting. What are the first things that come to mind?
2. Think about the challenges or frustrations faced by disabled people when they try to vote. What are the first things that come to mind?
3. How is this experience different for disabled people who are live in congregate settings as compared to those who live in a community?

*Probes*

- 1. *What kinds of challenges or frustrations do disabled people face when voting in person (e.g., physical access, technology, interactions with poll workers)?*
  2. *What kinds of challenges or frustrations do disabled people face when voting using absentee ballots?*

In what other parts of life do you think disabled people experience challenges or frustrations?

*[For each domain participants come up with, go through the following questions:]*

1. Think about the challenges or frustrations disabled people face when [insert participant stated domain]. What are the first things that come to mind?
2. In what ways do you think disabled people are not treated the same as non-disabled people in [insert participant stated domain]?
3. How are the challenges or frustrations in [insert participant stated domain] different for those who are disabled from a young age than those who become disabled when they are older?
4. Why do you think disabled people are not treated the same as non-disabled people in/at [insert participant stated domain]?
5. How do you think other identities and experiences shape a disabled person’s experience in [insert participant stated domain]?

**Critical Incident Technique**

1. Tell me about a time when you felt like you were treated unfairly because of your disability.
   1. Can you tell me what happened?
   2. Can you tell me how you felt?
   3. Can you tell me why you think you were treated unfairly?
   4. What do you wish had happened instead?
2. Tell me about a time when you felt like you were treated with respect, and all of your disability needs were met.
   1. Can you tell me what happened?
   2. Can you tell me how you felt?
   3. Can you tell me why you think you were treated with respect?
3. Can you talk about a time where somebody preferred to deal with someone else rather than dealing with you directly?
   1. Can you tell me what happened?
   2. Can you tell me how you felt?
   3. Can you tell me why you think they preferred to deal with someone else rather than you?
   4. What do you wish had happened instead?

***Thank you for taking the time to talk to us today. Your answers will help us understand how disabled people experience unfair treatment so that we can find ways to make sure it does not happen as often.***

***At the end of the study, we will share our results with the disability community and with other researchers. Do you want other people to know that you were a part of this study?***

**If participant says YES:**

***We can do that in two ways: 1) we can use your name next to your words. 2) we can let people know you were a part of this study without saying which words were yours.***

**If participant says NO:**

***We will make sure that your names stays private.***
