# Supplementary material for: Developing and Validating Measures of Structural Ableism to Improve Health Outcomes for the Disability Community: Protocol for a Mixed Methods Study
Source: JMIR Res Protoc. 2026 Mar 13;15:e86976. doi: 10.2196/86976 (PMC13032091; doi:10.2196/86976)
Supplement: Multimedia Appendix 5 [file resprot_v15i1e86976_app5.docx]

<https://youtu.be/H-OdbjYpoNM?si=lT_rB_D9uGI-HJSV>
